# Supplementary material for: Indocyanine Green (ICG) in Robotic Gastrectomy: A Retrospective Review of Lymphadenectomy Outcomes for Gastric Cancer
Source: Cancers (Basel). 2023 Oct 11;15(20):4949. doi: 10.3390/cancers15204949 (PMC10605643; doi:10.3390/cancers15204949)
Supplement: Supplementary file 1 [file cancers-15-04949-s001.zip › cancers-2631036-supplementary.pdf]

Table 1. Proper lymphadenectomy achievement by intervention group.

Variables, n (%)	PL (-)	PL (+)	p-value
**Intervention group**			
G1 vs G2	69 (24.0%) vs 12 (19.7%)	219 (76.0%) vs 49 (80.3%)	0.471
G2 vs G3	12 (19.7%) vs 4 (9.1%)	49 (80.3%) vs 40 (90.9%)	0.137
G3 vs G1	4 (9.1%) vs 69 (24.0%)	40 (90.9%) vs 219 (76.0%)	0.027

PL, proper lymphadenectomy

Table 2-1. Multivariate analysis of factors contributing to proper lymphadenectomy between intervention group (G1 vs G2).

Variables	Univariate			Multivariate		
	OR	95% CI	p-value	OR	95% CI	p-value
**Older age** (vs. < 65)	0.772	0.468-1.274	0.312			
**Male sex** (vs. Female)	0.966	0.567-1.646	0.898			
**ECOG 2-4** (vs. 0-1)	0.713	0.243-2.086	0.536			
**Higher BMI** (vs. <23)	0.966	0.581-1.609	0.895			
**No comorbidity** (vs.present)	0.124	0.359-1.132	0.638			
**No history of abdominal surgery** (vs. present)	0.685	0.393-1.195	0.183			
**TG (**vs. STG)	0.566	0.288~1.111	0.098			
**T stage**						
T1	Ref			Ref		
T2	2.240	0.944-5.315	0.067	1.572	0.592-4.177	0.364
T3	2.329	1.125-4.822	0.023*	2.491	1.031-6.019	0.043*
T4	2.688	1.157-6.246	0.022*	2.419	0.972-6.016	0.057
**N stage**						

	N0	Ref			Ref		
	N1	2.074	1.016-4.236	0.045*	2.074	1.016-4.236	0.045*
	N2	0.914	0.489-1.709	0.779	0.914	0.489-1.709	0.779
	N3	3.879	1.560-9.646	0.004*	3.879	1.560-9.646	0.004*
**Intervention group**							
	G1	Ref			Ref		
	G2	1.287	0.647~2.557	0.472	1.214	0.596-2.474	0.593

OR, odds ratio; CI, confidence interval; ECOG, Eastern Cooperative Oncology Group performance status; BMI, body mass index; TG, total gastrectomy; STG, subtotal gastrectomy.

Table 2-2. Multivariate analysis of factors contributing to proper lymphadenectomy between intervention group (G2 vs G3).

Variables	Univariate			Multivariate		
	OR	95% CI	p-value	OR	95% CI	p-value
**Older age** (vs. < 65)	1.030	0.343-3.091	0.958			
**Male sex** (vs. Female)	1.788	0.470-6.802	0.394			
**ECOG 2-4** (vs. 0-1)			1.000			
**Higher BMI** (vs. <23)	1.378	0.455-4.174	0.571			
**No comorbidity** (vs.present)	0.642	0.215-1.917	0.427			
**No history of abdominal surgery** (vs. present)	1.256	0.325-4.847	0.741			
**TG (**vs. STG)	1.536	0.505-4.673	0.450			
**T stage**						
T1	Ref			Ref		
T2			0.999			0.999
T3			0.999			0.999
T4			0.999			0.999
**N stage**						

	N0	Ref			Ref		
	N1	2.250	0.593-8.532	0.233	3.567	0.752-16.914	0.109
	N2	1.875	0.490-7.179	0.359	2.049	0.444-9.452	0.358
	N3			0.998			0.998
**Intervention group**							
	G2	Ref			Ref		
	G3	2.449	0.733-8.181	0.146	2.075	0.549-7.850	0.282

OR, odds ratio; CI, confidence interval; ECOG, Eastern Cooperative Oncology Group performance status; BMI, body mass index; TG, total gastrectomy; STG, subtotal gastrectomy.

Table 2-3. Multivariate analysis of factors contributing to proper lymphadenectomy between intervention group (G1 vs G3).

Variables	Univariate			Multivariate		
	OR	95% CI	p-value	OR	95% CI	p-value
**Older age** (vs. < 65)	0.723	0.429-1.219	0.224			
**Male sex** (vs. Female)	0.922	0.532-1.596	0.771			
**ECOG 2-4** (vs. 0-1)	0.603	0.203-1.795	0.364			
**Higher BMI** (vs. <23)	1.058	0.622-1.798	0.836			
**No comorbidity** (vs.present)	0.590	0.324-1.075	0.085			
**No history of abdominal surgery** (vs. present)	0.779	0.436-1.394	0.401			
**TG (**vs. STG)	0.526	0.245-1.089	0.084			
**T stage**						
T1	Ref			Ref		
T2	1.826	0.771-4.324	0.171	1.408	0.525-3.779	0.496
T3	1.984	0.968-4.067	0.061	2.344	0.941-5.840	0.067
T4	2.538	1.068-6.030	0.035*	2.740	1.053-7.128	0.039*
**N stage**						

	N0	Ref			Ref		
	N1	1.990	0.922-4.297	0.080	2.001	0.922-4.342	0.079
	N2	0.972	0.509-1.855	0.931	0.949	0.464-1.824	0.876
	N3	4.048	1.619-10.124	0.003*	3.965	1.578-9.961	0.003*
**Intervention group**							
	G1	Ref			Ref		
	G3	3.151	1.088-9.120	0.034*	3.156	1.076-9.256	0.036*

OR, odds ratio; CI, confidence interval; ECOG, Eastern Cooperative Oncology Group performance status; BMI, body mass index; TG, total gastrectomy; STG, subtotal gastrectomy.
